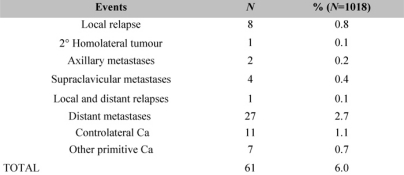# Management training for hospital administrators: sentinel lymph-node biopsy under local anaesthetic for carcinoma of the breast–organizational and economic impact

**DOI:** 10.3332/eCMS.2008.74

**Published:** 2008-02-15

**Authors:** V Galimberti, C De Cicco, P Veronesi, F Landoni, S Baraldi

**Affiliations:** European Institute of Oncology, Via G Ripamonti 435, I-20141, Milan, Italy

## Abstract

This study compares sentinel lymph-node biopsy carried out at the time of removal of the primary breast tumour, under general anaesthetic, with sentinel lymph-node biopsy carried out under local anaesthetic prior to the main operation. It compares the total cost of the two treatment approaches, in terms of average income and of their impact on the subsequent programming of operations and hence on waiting lists and income.

## Introduction

Sentinel lymph-node biopsy (SLNB) is a surgical technique used in the staging of breast cancer [1].

Various randomized clinical studies have compared the efficacy of sentinel lymph-node biopsy versus conventional axillary lymph-node dissection; at the moment the only published results are of a randomized clinical trial carried out at this institute, which confirm the accuracy of SLNB in assessing axillary lymph-node metastases, without increasing the risk of local relapse [2,3].

Furthermore, the thoroughness of the histological examination used to analyse the sentinel lymph-node (60 histological sections compared to three sections for the standard), indicated that this procedure was more reliable than the conventional histological examination, identifying axillary metastases in more cases (from 25–36% in T1 tumours) [4,5].

In view of these results, the SLNB procedure can be considered to all effects the standard clinical procedure (Evidence Based Medicine: level 1).

The large scale use of this technique will reduce the overall costs of treatment, in terms of shorter periods of hospitalization and a reduction in the complications associated with complete axillary lymph-node dissection.

In fact, the need for post-operative assistance, i.e. medication and physiotherapy, are notably reduced after this procedure when compared to complete axillary lymph-node dissection [6,7,8].

However, if the histological examination of the lymph node is carried out intra-operatively, about 20–60 minutes are required to obtain the extemporal result, prolonging the occupancy of the operating theatre and so hindering the programming of successive operations. Hence, in our institute, from September 2000, we have started to perform SLNB under local anaesthetic in day surgery, in patients with clear diagnosis of mammary carcinoma.

If the histological examination is definitive, and not extemporal, the SLN is evaluated before any successive operation to remove the primary neoplasia, which will be carried out under routine hospital recovery.

In 2002, we published the first clinical results from 115 patients, which confirmed the feasibility and safety of this procedure [9].

**Figure 1:**
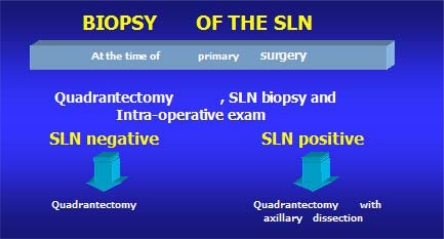


**Figure 2:**
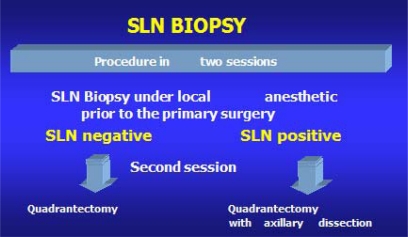


The aim of this study is to compare SLNB carried out at the time of the removal of the primary tumour under general anaesthetic (Figure 1) with SLNB carried out under local anaesthetic prior to the main operation under general anaesthetic (Figure 2).

The aim is to compare the total cost of the two treatment approaches, in terms of average income and of their impact on the subsequent programming of operations and hence on waiting lists and income.

## Materials and methods

From September 2000 to December 2005, within the European Institute of Oncology of Milan, 1018 patients with mammary carcinoma at stage cT1–cT2N0 (age range 27–83) were given a SLNB under local anaesthetic; all the patients, following analysis of the SLN, underwent treatment for the primitive neoplasia.

All the patients eligible for SLNB were treated in this manner, with the exception of obese patients or those with tumour progression into the axillary region. The median follow-up period was 19.7 months (range 1.0–76.0).

As this is a diagnostic procedure aimed at staging, the SLNB is carried out prior to the main surgical intervention to the breast and is carried out under local anaesthetic in day hospital. The possibility of programming the main surgical event after the result of the SLNB allows for a drastic reduction in the recovery period and hence in both the direct and indirect costs associated with the procedure.

## Surgical techniques

Radio-guided surgery is easy to use and does not require specific surgical training for surgeons already expert in oncological breast surgery.

The technique of SLNB under local anaesthetic does not differ substantially from that carried out under general anaesthetic. The surgical incision is just a few centimetres long and follows a natural axillary skin fold, within the hypothetical ‘italics’ incision line used for axillary dissections. It is always important to consider successive surgical treatments to the breast, and so if the neoplasia is located in the super-external quadrant, a quadrantectomy with an eventual axillary dissection will be carried out. In this case, the ideal incision will be along the lateral edge of the large pectoral muscle.

## Results

The definitive histological examination of the tumours of those patients who underwent an SLNB under local anaesthetic demonstrated that the SLN was negative for metastases in 595 cases (58.4%) and had ‘isolated tumour cells’ (ITC) pN0 (+ic) in 48 cases (4.7%). The remaining 18% had micro-metastases in the SLN (Table 3); of these approximately half took part in a randomized study in which no surgical treatment to the axilla was compared with axillary dissection. Hence, in total, 72.1% of the patients did not undergo successive axillary dissection.

The definitive histological examination indicated macro-metastases of the SLN in only 18.9% of the cases, and these patients had axillary dissection during the subsequent operation. The number of axillary relapses (0.2%) (Table 4) was less than expected (8%). The economic outcome is evaluated by analysing in detail the direct costs of the procedure (materials and medicines), the indirect costs, the cost of personnel, of the recovery and the cost of the pathology laboratory. The SLNB carried out under local anaesthetic costs €509; this value must be considered as part of the complete procedure, carried out as a standard recovery, as the income will be added to that of the standard recovery budget.

If the SLNB is performed together with the quadrantectomy under general anaesthetic, (Table 1, Treatment 1A), it has a total cost of €2268, offset by an income of €2925, leaving a margin of €657.

If instead the SLNB is performed under local anaesthetic with a successive quadrantectomy in standard recovery (Table 2, Treatment 2A), the total cost is €1796 offset by an income of €2925, leaving a margin of €1129.

Treatment 1B (Table 1), i.e. quadrantectomy plus SLNB and axillary dissection has a total cost of €3346 offset by an income of €4596, leaving a margin of €1250.

If instead the SLNB is performed under local anaesthetic with a successive quadrantectomy and axillary dissection in standard recovery (Treatment 2B, Table 2), the total cost is €2866 offset by an income of €4596, leaving a margin of €1730.

From an economic point of view, Treatment 1, SLNB under general anaesthetic, had higher indirect costs with respect to Treatment 2, i.e. SLNB under local anaesthetic, and some of the direct costs were also higher: operating room personnel and recovery. As the income is the same for the two procedures, the margin is 42% higher if the SLN is negative, and 28% higher if the SLN is positive.

## Conclusions

The use of SLNB is well established, and the procedural and economic differences between the two treatments are made evident here. From an economic point of view, carrying out a definitive histological examination of the SLN as opposed to an extemporal one allows a reduction in the time of the operation, meaning that more surgical interventions can be programmed successively, so reducing waiting lists and pathology laboratory expenses. By carrying out the SLNB under local anaesthetic, more precise programming of the definitive surgical treatment can be made in at least 60% of the cases so reducing the waiting list.

From this analysis, it also emerges that in cases that undergo axillary dissection due to SLN positivity in approximately 50% of cases the SLN is the only node affected. In these cases, axillary dissection of the histologically negative nodes does not influence the weight of the DRG as the initial positivity of the SLN is not taken into consideration. For the same procedure carried out under general anaesthetic, the positivity of a single SLN affects positively the weight of the DRG. This is the only real economic disadvantage of carrying out SLN under local anaesthetic.

## Figures and Tables

**Table 1:**
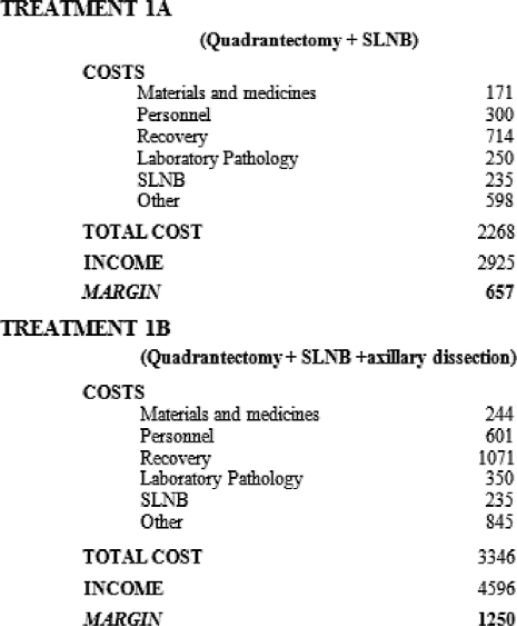
SLNB during primary surgery

**Table 2:**
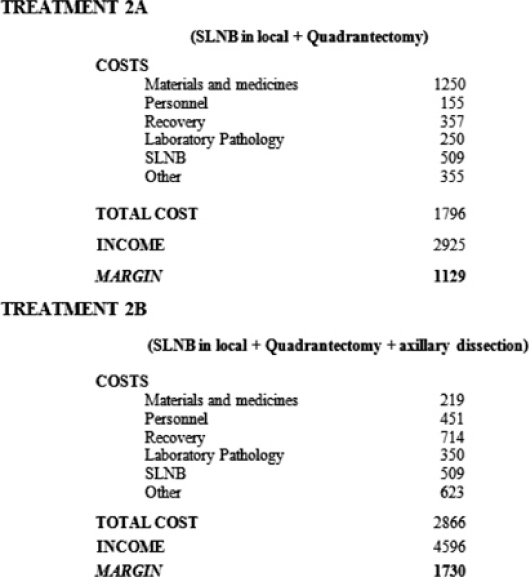
SLNB in day hospital (local anaesthetic)

**Table 3:**
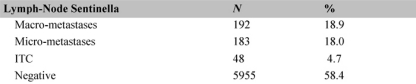
Histology of the SLN in 1018 cases

**Table 4:**